# Common gene expression patterns are observed in rice roots during associations with plant growth-promoting bacteria, *Herbaspirillum seropedicae* and *Azospirillum brasilense*

**DOI:** 10.1038/s41598-022-12285-3

**Published:** 2022-05-25

**Authors:** Grant Wiggins, Jacklyn Thomas, Yasir Rahmatallah, Connor Deen, Allee Haynes, Zachariah Degon, Galina Glazko, Arijit Mukherjee

**Affiliations:** 1 Department of Biology, University of Central Arkansas, Conway, AR 72035 USA; 2 Department of Biomedical Informatics, University of Arkansas for Medical Sciences, Little Rock, AR 72205 USA

**Keywords:** Molecular biology, Plant sciences, Plant molecular biology, Plant symbiosis

## Abstract

Non-legume plants such as rice and maize can form beneficial associations with plant growth-promoting bacteria (PGPB) such as *Herbaspirillum seropedicae* and *Azospirillum brasilense*. Several studies have shown that these PGPB promote plant growth via multiple mechanisms. Our current understanding of the molecular aspects and signaling between plants like rice and PGPB like *Herbaspirillum seropedicae* is limited. In this study, we used an experimental system where *H. seropedicae* could colonize the plant roots and promote growth in wild-type rice. Using this experimental setup, we identified 1688 differentially expressed genes (DEGs) in rice roots, 1 day post-inoculation (dpi) with *H. seropedicae*. Several of these DEGs encode proteins involved in the flavonoid biosynthetic pathway, defense, hormone signaling pathways, and nitrate and sugar transport. We validated the expression pattern of some genes via RT-PCR. Next, we compared the DEGs identified in this study to those we previously identified in rice roots during associations with another PGPB, *Azospirillum brasilense*. We identified 628 genes that were differentially expressed during both associations. The expression pattern of these genes suggests that some of these are likely to play a significant role(s) during associations with both *H. seropedicae* and *A*. *brasilense* and are excellent targets for future studies.

## Introduction

Plants can form mutually beneficial associations with a vast range of soil microbes, including arbuscular mycorrhizal fungi (AMF), soil bacteria rhizobia, and plant growth-promoting bacteria (PGPB)^1^. PGPB include a diverse array of microbes (*Azospirillum*, *Herbaspirillum*, *Burkholderia*, etc.) found in the rhizosphere, on plant root surfaces, or inside plant roots. These bacteria promote plant growth and can protect them against biotic and abiotic stresses. The mechanisms by which PGPB promote plant growth include biological nitrogen fixation (BNF), hormone synthesis, and stress reduction, among others^1–3^. Identifying the signaling pathways governing the associations between plants and different PGPB can play an important role in improving agricultural production sustainably.

Genetic studies in model legumes such as *Medicago truncatula* and *Lotus japonicus* have identified several plant genes required to establish legume-rhizobia symbiosis and AM symbiosis^4–6^. Some of these genes are part of the ‘common symbiotic pathway’ and are required for multiple plant–microbe symbioses^4,7^. Overall, these studies have contributed immensely to our knowledge of how legumes interact with different symbiotic partners. However, our current understanding of the host genes and signaling pathways regulating the associations between plants and different PGPB is still in its infancy. For instance, very few studies have investigated the regulation of gene expression occurring in host plants during associations with PGPB^8–12^. Previously, we performed a transcriptomic study to identify differentially expressed genes in rice roots upon inoculation with *Azospirillum brasilense,* one of the best-characterized PGPB^13^. We identified genes involved in the flavonoid biosynthetic pathway, defense, hormone signaling pathways, and nitrate and sugar transport, among others^13^. Further studies investigating the underlying molecular mechanisms between host plants and other PGPB will help improve our understanding of these beneficial plant–microbe associations.

*Herbaspirillum seropedicae* is a well-known PGPB that colonizes and benefits non-legume plants such as rice, sorghum, sugarcane, and maize^14–18^. Past studies have shown that different strains (e.g., B501, Z67) of *H. seropedicae* can promote plant growth via nitrogen fixation^15,19–21^. In the current study, we used the same experimental conditions from our previous study in *A. brasilense*^13^ to determine if *H. seropedicae* B501 could colonize rice roots and promote growth. Following that, we performed transcriptional profiling via RNA-seq to identify plant genes and pathways involved in the rice-*H. seropedicae* association. Since both *H. seropedicae* and *A. brasilense* promote plant growth via similar mechanisms, we anticipated that there would be an overlap in the expression profile of some genes during both associations. So, we compared the two datasets and identified common gene expression patterns in rice during its associations with these PGPB. The current study expands on past findings and will be an excellent resource for future studies investigating associations between rice and PGPB.

## Materials and methods

All experiments in this study were performed in accordance with the relevant guidelines and regulations of the journal.

### Plant material and growth conditions

We utilized wild-type rice (*Oryza sativa* cv. Nipponbare) seeds for all the experiments in this study. The plant preparation and growth conditions were similar to our earlier study^13^. The rice seeds were surface sterilized before germination. Next, the germinated seedlings were transferred to 15-cm Petri plates (#639102, Greiner bio-one, North Carolina, USA) containing low-N_2_ Fahraeus medium. These were grown for approximately 5–7 days in a Percival growth chamber (#CU-22L, Iowa, USA) with 150–200 μmol m^−2^ s^−1^ light intensity and relative humidity of 65% before bacterial inoculation.

### Bacterial inoculation, bacterial counts, and bacterial genotyping

The rice roots were inoculated with a *gfp*-tagged strain of *H. seropedicae* B501^20^ as described in our previous study^13^. Briefly, *H. seropedicae* B501 was grown on Tryptone Yeast-Extract (TY) media with Kanamycin at 30ºC to an optical density (600 nm) of 0.6. Prior to inoculation, the bacterial cells were resuspended in sterile water. The control rice seedlings were inoculated with sterile water, while the bacteria-inoculated seedlings were treated with 10^8^ cells/ml of *H. seropedicae.* Following inoculation, the plants were grown in a growth chamber as described above. The root colonization studies using plate count assays were performed as described in our previous study^13^. For bacterial genotyping, we extracted DNA from uninoculated and inoculated rice plants using Qiagen DNeasy® Plant Mini Kit (Cat # 69104, California, USA) according to the manufacturer’s guidelines. Next, we performed PCR using gene-specific primer sets to target the *nifH* and *gfp* genes, respectively. We used the rice *Cyclophilin* gene as a control in this experiment. These primers were used in earlier studies^22,23^ and are included in Supplementary Table 1. The PCR products were analyzed via electrophoresis on 2% (w/v) agarose gel. The gel is a representation of at least two biological replicates of each sample.

### RNA extraction, RNA sequencing, and data analysis

Total RNA was extracted from the plant roots at one-day post-inoculation using Qiagen RNeasy® Plant Mini Kit (Cat #74904, California, USA), as mentioned in^13^. Three biological replicates were utilized for each sample, inoculated and uninoculated. Quantification of RNA, library preparation, and sequencing were performed at the Research Technology Support Facility (RTSF), Michigan State University, East Lansing, MI, USA. RNA integrity was checked via a Bioanalyzer (Agilent Technologies) prior to sequencing library preparation using Illumina TruSeq Stranded mRNA Library Preparation Kit. The completed libraries were quality controlled, then quantified using Qubit dsDNA HS, Caliper LabChipGX HS DNA, and Kapa Illumina Library Quantification qPCR assays. All of the libraries were pooled in equimolar quantities, and this pool was loaded onto one lane of a HiSeq 4000 flow cell and sequencing a 2 × 150 bp paired-end format using HiSeq reagents. Base calling was performed by Illumina Real-Time Analysis (RTA) v2.7.6, and the output of RTA was multiplexed and converted to FastQ format with Illumina Bcl2fastq v2.18. Raw paired-end sequencing data is available from the Sequence Read Archive (SRA) under the BioProject accession number PRJNA525147. Raw paired-end reads in the FastQ files were examined for possible low base score, Illumina adapter, and PCR contaminations using *fastQC*. Illumina TruSeq adapter sequences were detected in both forward and reverse reads, and Illumina Single End PCR Primer sequences were detected in reverse reads. We used *Trimmomatic*^24^ to (1) remove Illumina TruSeq adapter and PCR primer sequences, (2) remove leading and trailing bases with low quality score, (3) scan each read with a 4-base wide sliding window and cut from the position with average quality per base below 15 to the end, and (4) drop reads shorter than 36 bases long after cutting low quality ends. Paired-end reads were then processed using the Tophat-Cufflinks pipeline^25^ to obtain normalized gene expression profiles. We mapped the reads to the rice genome (*Oryza sativa*) using *Tophat* (v2.0.12)^26^, allowing two base pair mismatches per read (default parameter). We downloaded the genome contigs (file *all.chrs.con*), gene annotations (file *all.gff3*) for 55,986 loci, and short descriptions (file *all.locus_brief_info.7.man*) of the Michigan State University (MSU) *Oryza sativa* genome model (version 7) from the Rice Genome Annotation Project^27^. Aligned reads to annotated loci were quantified and normalized (FPKM normalized values) using *cufflinks* (v2.2.1)^28^. The normalized FPKM gene expression matrix is available in Supplementary Table 2. We performed differential expression analysis using cuffdiff (part of the Cufflinks suite) and identified the significantly differentially expressed genes (DEGs) as those with false discovery rate (FDR) < 0.05 and absolute fold-change greater than 2 (|FC|> 2 or |log_2_FC|> 1).

### Gene expression validation

We validated the results of RNA-sequencing for some genes via reverse-transcriptase PCR (RT-PCR) as described in Thomas et. al. Before cDNA synthesis, we treated the RNA samples with Ambion® DNA-free™ DNase Treatment and Removal kit (Cat #AM1906, California, USA). Next, we synthesized first strand cDNA from 300 ng of RNA using a Thermo Scientific RevertAid RT kit (Cat #K1691, Delaware, USA) with Oligo(dT)_18_ primers according to manufacturer’s instructions. RT-PCR was performed in three biological replicates for all the samples. We used *Cyclophilin* as an internal reference gene for the RT-PCR analysis^13,23^. The list of genes and their corresponding primer sequences are listed in Supplementary Table 5.

## Results

### *Herbaspirillum seropedicae* increased rice growth and colonized plant roots

**Figure 1:**
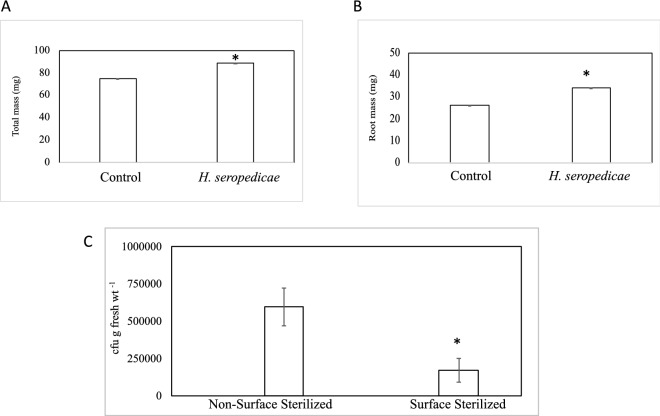
Growth promotion and root colonization in wild-type rice plants by *H. seropedicae*. (**A**) Shows that the total plant mass of wild-type rice plants increased significantly upon inoculation with *H. seropedicae*. Asterisk (*) denotes a significant difference between the two treatments by *t*-test (*P* < 0.002). Data represent the average of three experimental replications (n = 15) ± SE. (**B**) Shows that the root mass of wild-type rice plants increased significantly upon inoculation with *H. seropedicae.* Asterisk (*) denotes a significant difference between the two treatments by *t*-test (*P* < 0.01). Data represent the average of three experimental replications (n = 15) ± SE. (**C**) Shows a comparison of total colony-forming units (cfu) of *H. seropedicae* determined by serial dilution and plate counts of bacteria between non-surface sterilized and surface-sterilized roots of wild-type rice seedlings inoculated with *H. seropedicae*. The data are an average of three experiments. Each experiment had at least three plants. Asterisk (*) denotes a significance between the conditions by *t*-test (*P* < 0.001).

We wanted to determine if *Herbaspirillum seropedicae* could increase growth in wild-type rice under our experimental conditions. We found that the total plant mass was 1.2-fold higher in *H. seropedicae*-inoculated plants than in the uninoculated plants (Fig. 1A). We also found that the average root mass was 1.3-fold higher in *H. seropedicae-*inoculated plants compared to the uninoculated plants (Fig. 1B). To confirm the presence of *H. seropedicae* in the inoculated samples, we used PCR to amplify fragments of *nifH* and *gfp* genes, both specific to the *gfp*-tagged strain of *H. seropedicae*, from these samples (Supplementary Fig. 1). As expected, the uninoculated samples did not yield any PCR products for these genes. We used the rice *Cyclophilin* gene as a control in this experiment. Next, using plate count assays, we determined whether *H. seropedicae* could colonize rice roots under these experimental conditions. Our results indicate that *H. seropedicae* could penetrate rice roots as we could retrieve bacterial colonies from the surface-sterilized plant roots (Fig. 1C).

### Rice root transcriptome analysis post-inoculation with *H. seropedicae*

**Figure 2:**
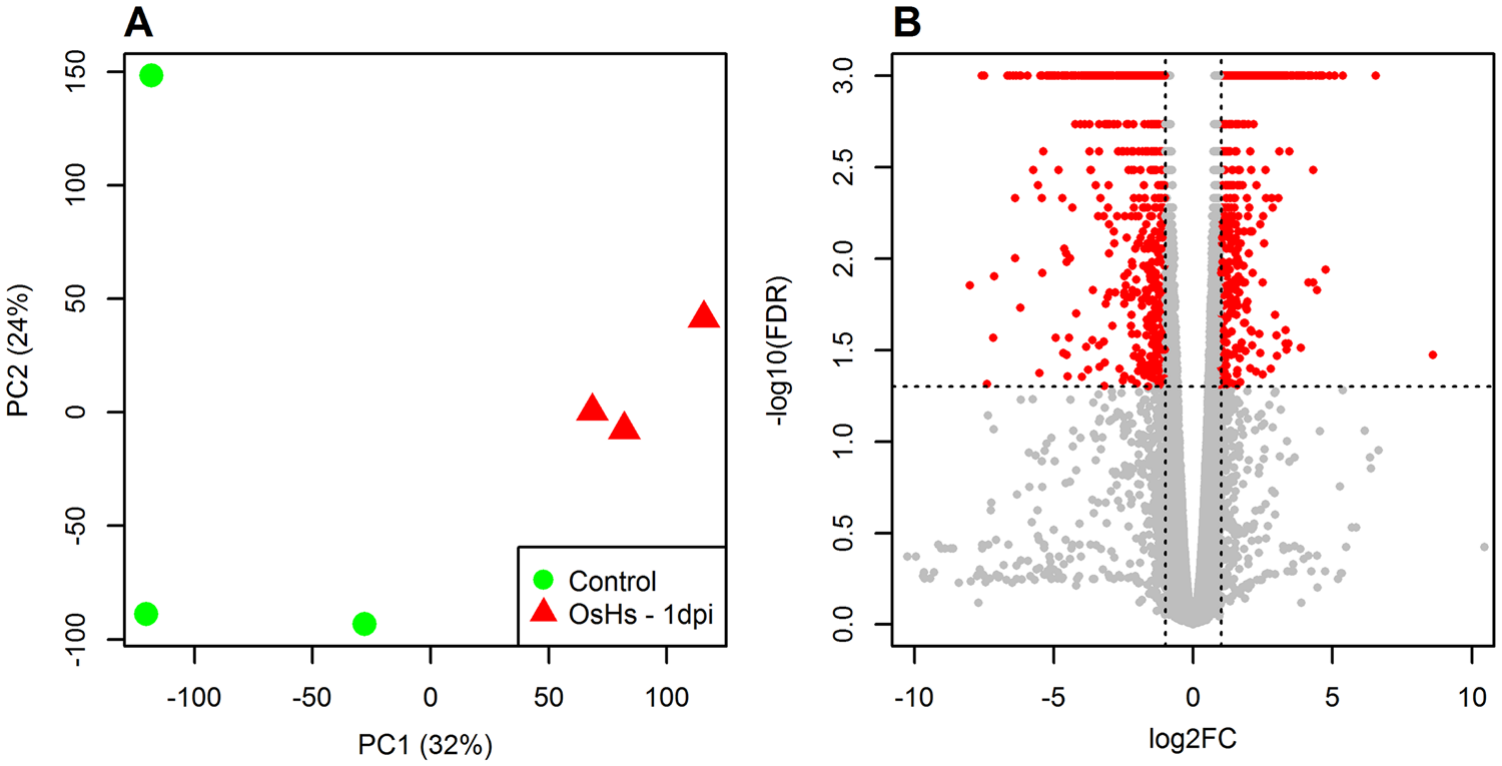
Summary plots for the gene expression profiles and differential expression analysis. (**A**) Shows a scatter plot of the two principal components of the FPKM normalized gene expression profiles. (**B**) Shows a volcano plot where mean log_2_fold change is plotted against the − log_10_ FDR adjusted P-values for all the expressed genes. Significant differentially expressed genes (FDR < 0.05 and |log_2_FC|> 1) are highlighted in red.

We utilized high-throughput RNA sequencing to identify differentially expressed genes (DEGs) in rice roots post-inoculation with *H. seropedicae*. Our samples for the RNA sequencing experiment included: wild-type roots + *H. seropedicae* (1-day post-inoculation (dpi)) vs. wild type roots + mock treatment (water only), at the same time point (1 dpi). Each sample included three biological replicates. The sequencing libraries were prepared from these RNA samples, quality checked and quantified prior to sequencing in a 2 × 150 bp paired-end format using HiSeq 4000. We obtained an average of 38 million raw reads per sample and an average of 23 million filtered reads per sample. The filtered reads had an average mapping rate of 85% to the rice genome (MSU, version 7) (Supplementary Table 3). We observed a good degree of correlation between the different biological replicates of each sample using two-dimensional principal component analysis scatter plot (Fig. 2A). Next, we identified the DEGs from our dataset using an FDR adjusted P-value of < 0.05 and a fold change (FC) of > 2 (|Log_2_FC|) > 1). The total number of DEGs identified in *H. seropedicae-*inoculated rice roots was 1688. Among these 856 genes were upregulated in expression and 832 genes were downregulated in expression (Fig. 2B, Supplementary Table 4).

**Figure 3:**
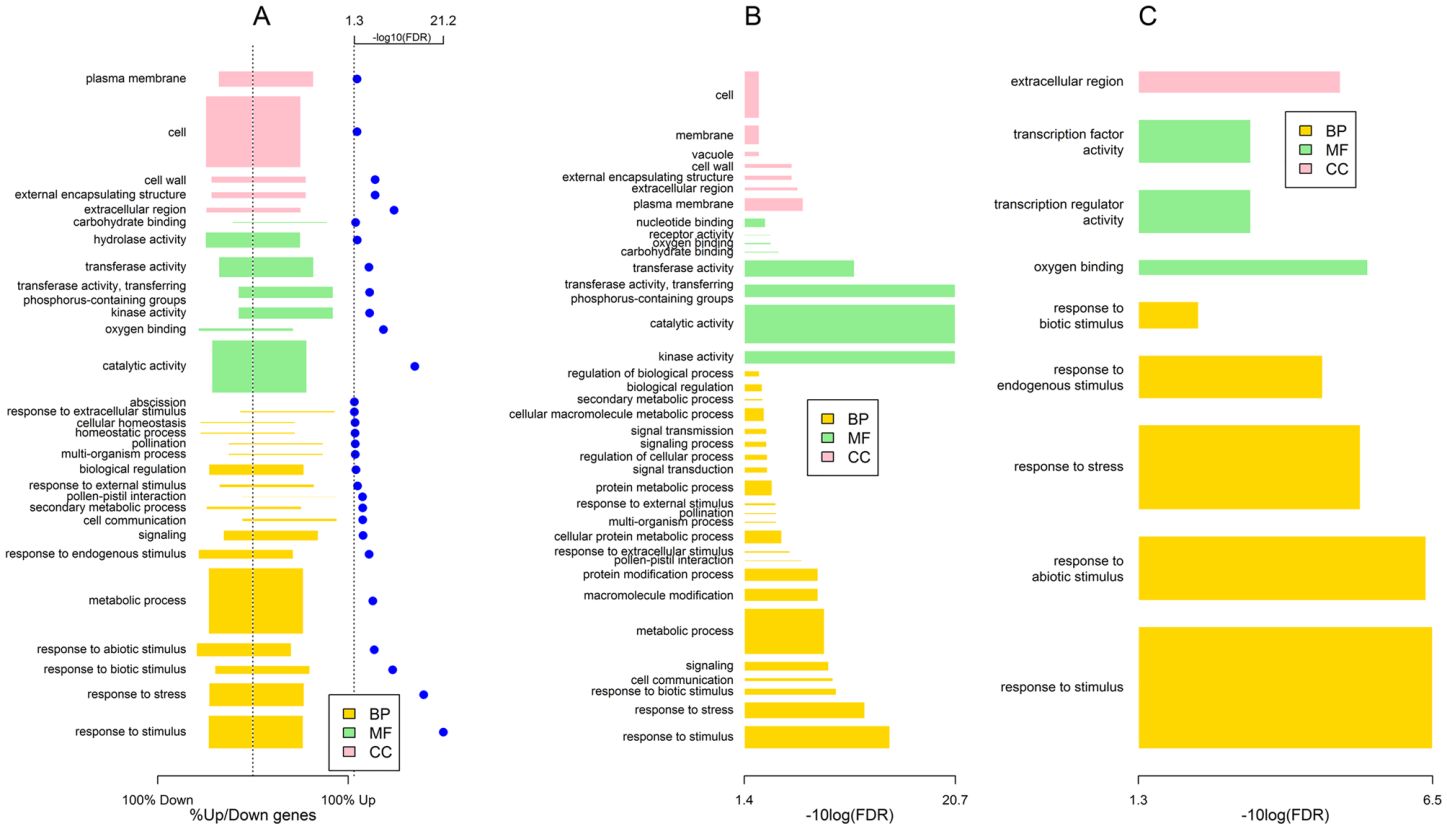
Bar plots summarizing the gene ontology terms over-represented in the DEGs when considering all 1688 DEGs (**A**), 856 upregulated DEGs (**B**), and 832 downregulated DEGs (**C**). In (**A**), the horizontal axis indicates the proportion of up- and down-regulated genes among the DEGs associated with each GO term, where the bar ends indicate these proportions. In (**A**), the blue dots represent − log_10_(FDR) for each significant GO term. A vertical dotted line at − log_10_(0.05) = 1.3 indicates the significance threshold. In (**B**,**C**), the bars indicate the significance level (− log_10_(FDR)) of each GO term associated respectively with the upregulated and downregulated DEGs. The width of each bar in all the figures is proportional to the number of DEGs associated with the GO terms. Bar color indicates the three categories of GO terms: *BP* biological processes, *MF* molecular functions, *CC* cellular components.

Next, we performed gene ontology (GO) analysis to understand the biological significance of the DEGs. Using singular enrichment analysis (SEA) with agriGO^29^, we identified 30 GO terms that were significantly enriched for the 1688 DEGs. There were eighteen terms in biological processes (e.g., response to stimulus, response to stress, signaling), seven terms in molecular functions (e.g., catalytic activity, kinase activity), and five terms in the cellular component (e.g., extracellular region, cell wall) (Fig. 3A). For the 856 upregulated genes, we identified thirty-eight GO terms (Fig. 3B). These included twenty-three GO terms in biological processes (e.g., response to stimulus, response to stress, signaling), eight GO terms in molecular functions (e.g., catalytic activity, kinase activity, receptor activity), and seven GO terms in the cellular component (e.g., plasma membrane, extracellular region, cell wall) (Fig. 3B). For the 832 downregulated genes, we identified nine GO terms, with five GO terms in biological processes (e.g., response to stimulus, response to stress), three GO terms in molecular functions (oxygen binding, transcription factor activity, and transcription regulator activity), and one GO term in the cellular component (extracellular region) (Fig. 3C).

Past studies have shown that genes belonging to the flavonoid synthetic pathway, plant defense, and hormone signaling play essential roles during plant–microbe associations^6,13,30–34^. Genes encoding for transcription factors, protein kinases, and transporters are also critical components in the signaling pathways regulating these associations. In our list of 1688 DEGs, we focused on these gene classes that have a likelihood of playing a role during rice- *H. seropedicae* associations. Among genes belonging to the flavonoid synthesis pathway, we identified naringenin synthesis genes, chalcone synthesis genes, phenylalanine ammonia-lyase genes, flavonol synthase genes, and isoflavone reductase to be differentially expressed. Several genes belonging to plant defense signaling (e.g., pathogenesis-related genes, thionin genes, chitinases, and cinnamoyl-CoA-reductase) were differentially expressed in rice roots. We identified several genes belonging to hormone signaling pathways (e.g., auxin-responsive (e.g., *SAUR*, *GH3*) genes, auxin-induced genes, auxin response factors, 1-aminocyclopropoane-1-carboxylate (ACC) oxidase genes) to be differentially expressed in our dataset. Transcription factors belonging to the AP2 family, helix-loop-helix, MYB family, NAC family, GRAS family, and WRKY family were well represented in our dataset. Among protein kinases, SHR5 receptor-like kinases, cysteine-rich receptor-like kinases, wall-associated kinases, serine-threonine receptor kinases, phytosulfokine receptors were differentially expressed in our dataset. As expected, our list of differentially expressed genes contained several transporters. These included high-affinity nitrate transporters, peptide transporters, sugar transporters, ammonium transporter, and nodulin genes. We also identified nitrate reductases that were differentially expressed in our dataset (Supplementary Table 4).

### Gene expression validation

**Figure 4:**
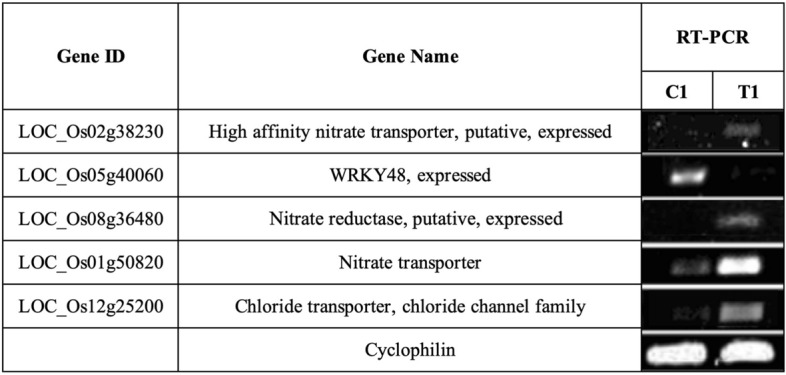
Validation of the expression pattern of five differentially expressed genes by RT-PCR. In these experiments, C1 and T1 represent cDNA synthesized from control and bacteria-inoculated samples. RT-PCR was performed in three biological replicates for all the samples, and *Cyclophilin* was used as an internal gene.

We examined the expression pattern of five randomly selected genes via reverse transcription polymerase chain reaction (RT-PCR) to confirm the gene expression patterns identified by the RNA-seq experiment (Fig. 4). We designed the primers used in these experiments based on the Rice Genome Annotation Project database annotations (Supplementary Table 5). The RT-PCR results confirm the expression patterns of these genes identified via RNA-seq (Fig. 4).

### Comparison of DEGs in rice roots during associations with *H. seropedicae* and *A. brasilense*

**Figure 5:**
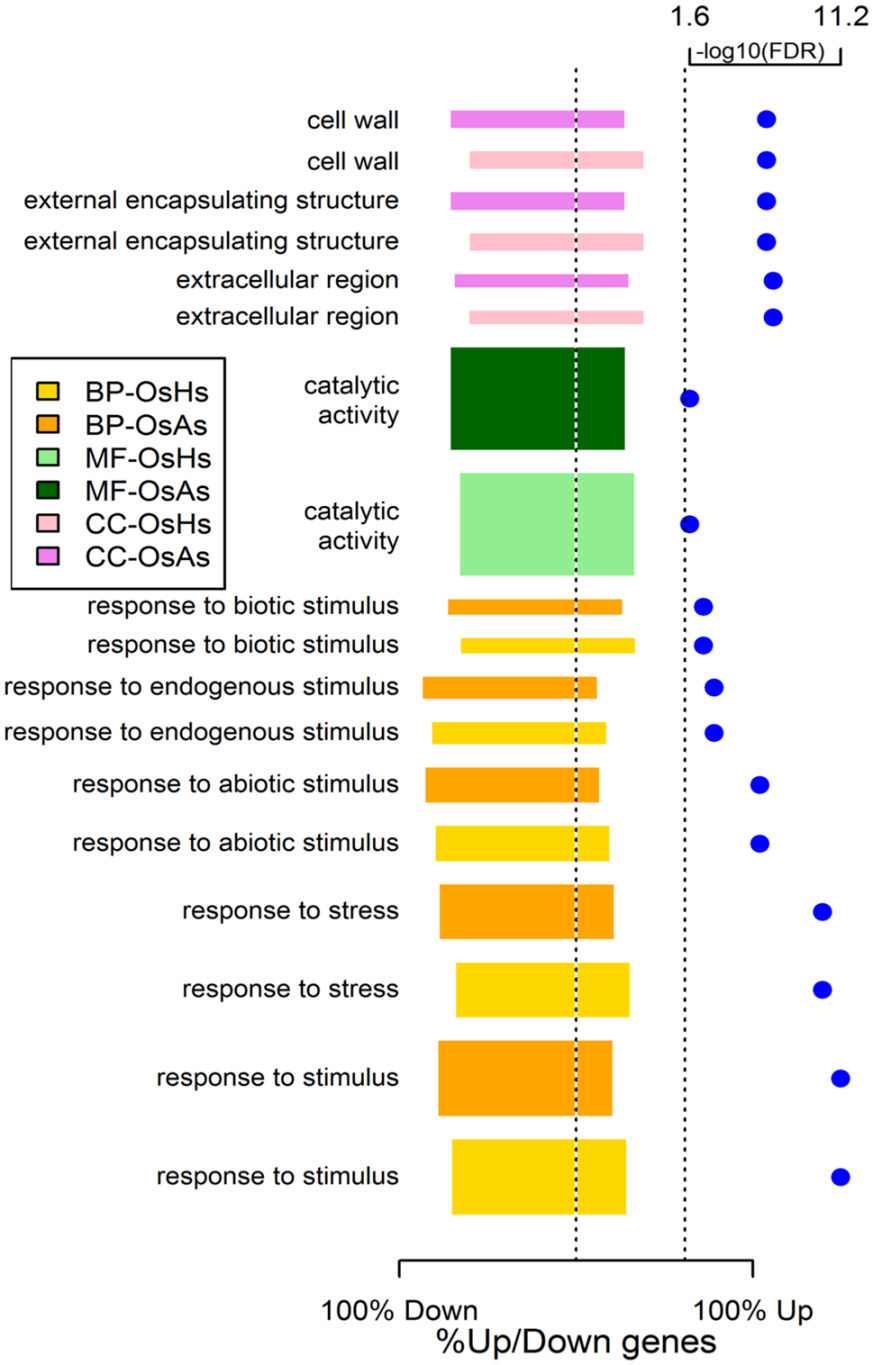
Bar plot summarizing the gene ontology terms over-represented in the 628 genes differentially expressed in both *H. seropedicae-* and *A*. *brasilense-*inoculated rice roots. Bar width is proportional to the number of genes associated with the corresponding GO term. The horizontal position of each bar around the center indicates the proportion of up-regulated and down-regulated DEGs associated with the corresponding GO term. Bar colors indicate the comparisons (*H. seropedicae*-inoculated (OsHs) and *A. brasilense*-inoculated (OsAs) rice roots against controls) and the three categories of GO terms: *BP* biological processes, *MF* molecular functions, *CC* cellular components. The blue dots represent − log_10_(FDR) for each significant GO term. The vertical dotted line at − log_10_(0.05) = 1.3 indicates the significance threshold.

Previously, we identified differentially expressed genes in rice roots during associations with *Azospirillum brasilense*^13^. In the rice-*A. brasilense* study, we analyzed the rice transcriptome 1 dpi with *A. brasilense* under similar experimental conditions used in the current study. In fact, the libraries from the different rice root samples (mock, *H. seropedicae*-inoculated, and *A. brasilense-*inoculated) were all sequenced in the same batch to avoid any possible batch effects when comparing the gene expression patterns in rice roots during associations with these two different PGPB. We identified 628 genes that were differentially expressed during associations with both bacteria (Supplementary Table 6). We performed GO analysis to understand the biological significance of these commonly expressed DEGs and identified 9 GO terms that were significantly enriched for the 628 DEGs (Fig. 5). There were five terms in biological processes (e.g., response to stimulus, response to stress, and response to endogenous stimulus), one term in molecular functions (catalytic activity), and three terms in the cellular component (e.g., extracellular region, cell wall, and external encapsulating structure) (Fig. 5). Some GO terms are more specific and associated with fewer genes than others. We also observe more down-regulated genes than upregulated genes in this common list of differentially expressed genes (Fig. 5). Specifically, among the 628 DEGs, 148 genes were upregulated, and 431 genes were downregulated in expression during both associations (Supplementary Table 6). Some of the genes differentially expressed by both bacteria included the flavonoid synthesis pathway genes (e.g., naringenin, flavonol synthase), receptor-like kinases (e.g., SHR5, OsWAK) and transporters (e.g., high-affinity nitrate, sugar, peptide, nodulins), among others. Among the downregulated DEGs, defense signaling genes (e.g., thionin, chitinases) and hormone-related genes (e.g., auxin-responsive genes, ACC oxidases) were well represented in the dataset (Supplementary Table 6).

## Discussion

Crops such as rice and maize can form beneficial associations with different plant growth-promoting bacteria (PGPB) such as *Azospirillum*, *Herbaspirillum*, *Azotobacter*, etc. These PGPB can promote plant growth via nitrogen fixation and phytohormone production^1^. However, our current understanding of the genetic pathways regulating the associations between host plants and PGPB is limited. In this study, we used an experimental setup in which the PGPB *Herbaspirillum seropedicae* could colonize rice roots and promote growth. Unlike legume-rhizobia endosymbiosis, these bacteria induce no specialized structures. Therefore, transcriptomic studies must collect data under appropriate experimental conditions where the plant perceives the bacterium as a beneficial partner. Previously, we used the same experimental setup for studies in rice-*A. brasilense* associations. These results further support the use of this experimental system for future studies investigating associations between rice and PGPB. Our next goal was to identify gene expression changes in rice roots upon inoculation with *H. seropedicae*. In this study, we focused on an early time point (1 day post-inoculation) to identify genes that might be required in the initial stages of this association.

Using RNA-sequencing, we identified 1688 differentially expressed genes in rice roots during associations with *Herbaspirillum seropedicae* (Supplementary Table 4). Next, we compared the DEGs identified in this study with the DEGs we had previously identified in rice roots during associations with *A. brasilense* and identified 628 genes differentially expressed in rice roots during associations with both bacteria (Supplementary Table 6). Below we discuss different classes of genes (e.g., flavonoid signaling, defense signaling, hormone signaling, transporters, etc.), which have a likelihood of playing significant roles during these beneficial rice-bacterial associations.

### Flavonoid synthesis genes

Flavonoids are secondary metabolites and are ubiquitous in plants. Studies have shown that flavonoids play critical roles during plant–microbe symbioses. Besides playing essential roles during legume-rhizobia symbiosis, arbuscular mycorrhizal symbiosis, and actinorhizal symbiosis, flavonoids are important regulators of other plant–microbe symbioses^30,35^. One study reported that flavonoids stimulate wheat root colonization by *Azospirillum brasilense* and *Azorhizobium caulinodans*^36,37^. The flavonoid naringenin also regulates the association between *H. seropedicae* and its host plants^37^. In the current study, we identified several genes from the flavonoid biosynthetic pathway to be differentially expressed in rice roots. These included chalcone synthase genes (LOC_Os07g34260 and LOC_Os11g32650), flavonol synthase genes (LOC_Os03g03034 and LOC_Os10g40900), and naringenin synthesis genes (LOC_Os04g49210 and LOC_Os04g49194). The chalcone synthase gene (LOC_Os07g34260), the flavonol synthase gene (LOC_Os03g03034), and the naringenin synthesis gene (LOC_Os04g49194) were also differentially expressed in rice roots during associations with *A. brasilense*^13^. A member of the isoflavone reductase (LOC_Os01g13610) gene family, an enzyme that catalyzes the biosynthesis of isoflavonoids, was upregulated in expression in rice roots. One study in *Phaseolus vulgaris* showed that transcript levels of an isoflavone reductase increased in roots during the early stages of interaction with *Rhizobium etli*^38^. Phenylalanine ammonia lyase (PAL) plays an important role in the biosynthesis of secondary plant metabolites, and *PAL* genes have been shown to be upregulated in expression during the early stages of arbuscular mycorrhizal symbiosis and legume-rhizobia symbiosis^39,40^. In this study, we observed two *PAL* genes (LOC_Os04g43800 and LOC_Os12g33610) that were upregulated in expression in rice roots. The *PAL* gene (LOC_Os04g43800) was also differentially expressed in rice roots during associations with *A. brasilense*^13^. The expression pattern of these flavonoid synthesis genes further suggests that flavonoids likely play key roles during beneficial rice-bacterial associations.

### Defense signaling genes

Several studies have reported that the host plant suppresses its defense mechanisms during beneficial plant–microbe associations^33,34,41,42^. This includes repression of defense-related gene (e.g., thionins, chitinases, pathogenesis-related genes, cinnamoyl-CoA-reductase, etc.) expression. In this study, we observed several thionin genes (LOC_Os06g31280, LOC_Os06g31800, LOC_Os06g32240, LOC_Os01g51270, etc.) downregulated in expression in rice roots. The thionin genes (LOC_Os06g31280 and LOC_ Os06g31800) were also suppressed in expression during rice-*A. brasilense* associations^13^. Thionin gene expression has also been shown to be differentially regulated in rice roots during associations with *H. seropedicae* in other studies^9,10^. We observed several chitinases (LOC_Os03g30470, LOC_Os04g41680, LOC_Os04g41620, etc.) that were downregulated in expression in rice roots. These chitinase genes were downregulated in expression during rice-*A. brasilense* associations as well^13^. The pathogenesis-related (*PR*) genes are excellent reporter genes for plant defense reactions. For instance, the *PR* gene (LOC_Os08g28670) was highly upregulated in expression in rice roots infected with the plant pathogen, *Magnaporthe oryzae*^43^. In this study, the same *PR* gene (LOC_Os08g28670) was downregulated in expression. In our previous rice-*A. brasilense* study, we observed one *PR* gene (LOC_Os12g36880) was downregulated in expression at 1dpi^13^. Interestingly, in this study, this gene (LOC_Os12g36880) was upregulated in expression. The cinnamoyl-CoA-reductase is also an excellent reporter gene for plant defense reactions. Some of these genes have also been shown to be repressed in expression during associations with mutualistic microbes^44–46^. In this study, we identified a cinnamoyl-CoA-reductase gene (LOC_Os08g17500) downregulated in expression in rice roots. Earlier, we had observed a different cinnamoyl-CoA-reductase gene (LOC_Os02g56700) suppressed in expression in rice roots during associations with *A. brasilense*^13^. Overall, we observe that some key defense-related genes are repressed in expression in rice roots during associations with *H. seropedicae*. The expression pattern of some of these genes is also conserved in plants during associations with other beneficial microbes, including *A. brasilense*.

### Hormone signaling genes

Plant hormones play critical roles in regulating different plant–microbe symbioses^6,31,32,47^. The hormone auxin has been studied the most and is an important regulator of plant–microbe symbioses^6,23,31,32,48^. Studies have shown that auxin regulates these associations by modulating the expression of a large number of genes, including auxin-responsive genes such as *SAUR*, *Aux/IAA*, *GH3*, etc.^49–51^. In our previous study with *A. brasilense*, we reported that several auxin-responsive genes were downregulated in expression in rice roots^13^. Other studies in *H. seropedicae* also showed a similar expression pattern of these auxin-responsive genes^9,10^. Here, we identified several auxin-responsive genes, including a *SAUR* gene (LOC_Os09g37480), *Aux/IAA* genes (LOC_Os02g56120, LOC_Os03g58350, etc.), a *GH3* gene (LOC_Os01g57610) that were downregulated in expression. Further studies can investigate how auxin regulates these associations. Ethylene is another major plant hormone critical to several plant processes, including beneficial plant–microbe associations^6,32,47^. The 1-aminocyclopropane-1-carboxylate (ACC) oxidase catalyzes important steps in ethylene synthesis^52^. In this study, we identified two ACC oxidase genes (LOC_Os09g27820 and LOC_Os08g30080) that were downregulated in expression. Expression of these genes was also suppressed in rice roots during associations with *A. brasilense*^13^. Our transcriptome data further suggest that the repression of these hormone pathways might be necessary for rice-bacterial associations.

### Other genes (transporters, transcription factors, receptor kinases, etc.)

In the current study, we identified several transporters that might be crucial to this association. Past studies have shown that nitrate transporters play essential roles in transporting nitrate, peptides, amino acids, and hormones, among others^53^. These genes have been shown to be key regulators of plant growth and development and nitrogen use efficiency. The nitrate uptake system in plants primarily consists of the high-affinity transporters and the low-affinity transporters^53,54^. In this study, we identified two high-affinity nitrate transporters (LOC_Os02g38230 and LOC_Os01g50820) that were upregulated in expression in rice roots. These genes were also highly expressed in rice roots during associations with *A. brasilense*^13^. This common expression pattern suggests an important role of these genes during these beneficial plant–microbe associations. We also identified low-affinity nitrate transporters like the peptide transporters (LOC_Os03g04570, LOC_Os01g54515, LOC_Os02g48570, etc.) that were differentially expressed in rice roots. Some of these transporters (e.g., LOC_Os03g04570, LOC_Os02g48570) were also differentially expressed during rice-*A. brasilense* associations, suggesting a similar role of these genes^13^. Ammonium, an important source of nitrogen for plants, is taken up by plant cells via ammonium transporters^55^. Studies have shown that these transporters play important roles during different plant–microbe symbioses^56^. In our rice-*A. brasilense* study, we had identified a few ammonium transporters that were differentially expressed in rice roots^13^. Here we identified one ammonium transporter (LOC_Os02g34580) that was differentially expressed in rice roots. In beneficial plant–microbe associations, sugar transporters have been shown to play key roles in the transfer of carbohydrates to the microbial symbionts^57–59^. In the rice-*A. brasilense* study, we identified one sugar transporter (LOC_Os04g37970) that was upregulated in expression in rice roots^13^. Here, we identified the same gene to be upregulated in expression during associations with *H. seropedicae*, suggesting a conserved role of this gene in these associations. We identified several nodulin genes (*early nodulin 93*, *MtN3*, *Major facilitator superfamily*, etc.) that were differentially expressed in rice roots. Some of these genes (e.g., LOC_Os10g34040, LOC_Os06g05010, LOC_Os03g22590) were also differentially expressed during associations with *A. brasilense*^13^. The role of these genes in beneficial rice-bacterial associations need to be explored further. As previously reported in our rice-*A. brasilense* study^13^, here also we identified several transcription factors and receptor kinases that were differentially expressed in rice roots. Some of the commonly expressed transcription factors identified include an auxin response factor (LOC_Os02g04810), a GRAS family transcription factor (LOC_Os12g04200), AP2 domain-containing transcription factors (LOC_Os02g42585, LOC_Os07g22770, LOC_Os04g57340), and several MYB family transcription factors (LOC_Os05g50350, LOC_Os08g33050, LOC_Os05g50340, LOC_Os02g40530, LOC_Os01g44390), among others. Similarly, we identified several receptor kinases that were commonly regulated in expression. These included SHR5 RLKs (e.g., LOC_Os05g17800, LOC_Os05g17604, LOC_Os08g10310), cysteine-rich RLKs (e.g., LOC_Os04g25060, LOC_Os04g25650), and wall-associated kinases (e.g., LOC_Os04g30060, LOC_Os04g30110, LOC_Os04g30250) among others. Further studies can investigate the roles of these genes in these beneficial plant-bacteria associations. In this study, we also identified two nitrate reductase genes (LOC_Os08g36480 and LOC_Os02g53130) that were differentially expressed in rice roots. These genes were also differentially expressed in rice roots during associations with *A. brasilense*^13^. The nitrate reductase enzyme plays a crucial role in nitrogen acquisition by plants^60^. Several studies have also reported high nitrate reductase expression in legume root nodules^61–65^. A recent study in *M. truncatula* showed plant nitrate reductases regulate nitric oxide production and nitrogen-fixing metabolism during the symbiotic association with *Sinorhizobium meliloti*^66^. Future studies can investigate the role of nitrate reductases during associations between plants and PGPB.

## Conclusions

**Figure 6:**
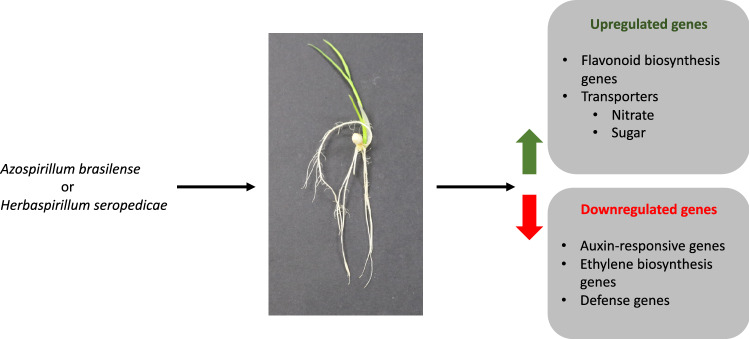
A model summarizing the common trends in gene expression changes observed in rice roots during associations with plant growth-promoting bacteria, *Azospirillum brasilense* and *Herbaspirillum seropedicae*.

In this study, we show that *Herbaspirillum seropedicae* can promote rice growth and penetrate its roots under our experimental conditions. We had previously used the same experimental system for studying the rice-*Azospirillum brasilense* association. Next, we used RNA-seq and identified the differentially expressed genes in rice during its association with *H. seropedicae*. Our transcriptome results suggest that flavonoids are likely involved in this association. We also report that the host plant’s defense responses are repressed, a pattern observed in other beneficial plant–microbe associations. Some genes from auxin and ethylene pathways were also suppressed in expression. Finally, we report the induction of nitrate and sugar transporters during the rice- *H. seropedicae* association. Interestingly, some of these gene expression trends were also conserved in the rice-*A. brasilense* association (Fig. 6), suggesting a shared pathway during these plant–microbe associations. Overall, this study will be an excellent resource for future studies investigating associations between rice and different PGPB.

## Supplementary Information

Supplementary Figure 1.

Supplementary Figure 2.

Supplementary Table 1.

Supplementary Table 2.

Supplementary Table 3.

Supplementary Table 4.

Supplementary Table 5.

Supplementary Table 6.
