# Supplementary material for: Common gene expression patterns are observed in rice roots during associations with plant growth-promoting bacteria, Herbaspirillum seropedicae and Azospirillum brasilense
Source: Sci Rep. 2022 May 25;12:8827. doi: 10.1038/s41598-022-12285-3 (PMC9132972; doi:10.1038/s41598-022-12285-3)
Supplement: Supplementary file 1 — Supplementary Figure 1. [file 41598_2022_12285_MOESM1_ESM.pdf]

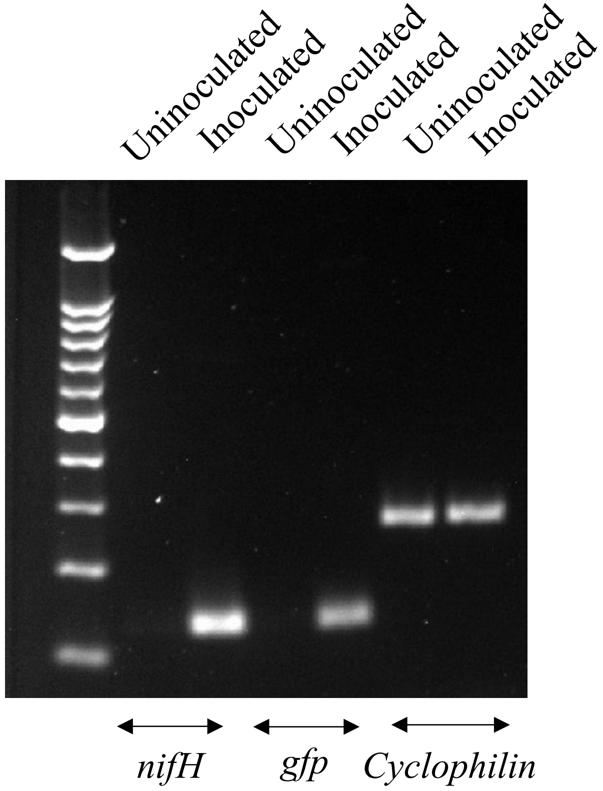

Supplementary Figure 1: Gel electrophoresis image confirming the presence of *H. seropedicae* in the inoculated samples. PCR was used to amplify fragments of *nifH* and *gfp* genes, both specific to the *gfp*-tagged strain of *H. seropedicae*, from inoculated and uninoculated samples. The rice *Cyclophilin* gene was used as a control in this experiment. The PCR products were analyzed via electrophoresis on 2% (w/v) agarose gel. The gel is a representation of at least two biological replicates of each sample.
