# Supplementary material for: Common gene expression patterns are observed in rice roots during associations with plant growth-promoting bacteria, Herbaspirillum seropedicae and Azospirillum brasilense
Source: Sci Rep. 2022 May 25;12:8827. doi: 10.1038/s41598-022-12285-3 (PMC9132972; doi:10.1038/s41598-022-12285-3)
Supplement: Supplementary file 2 — Supplementary Figure 2. [file 41598_2022_12285_MOESM2_ESM.pdf]

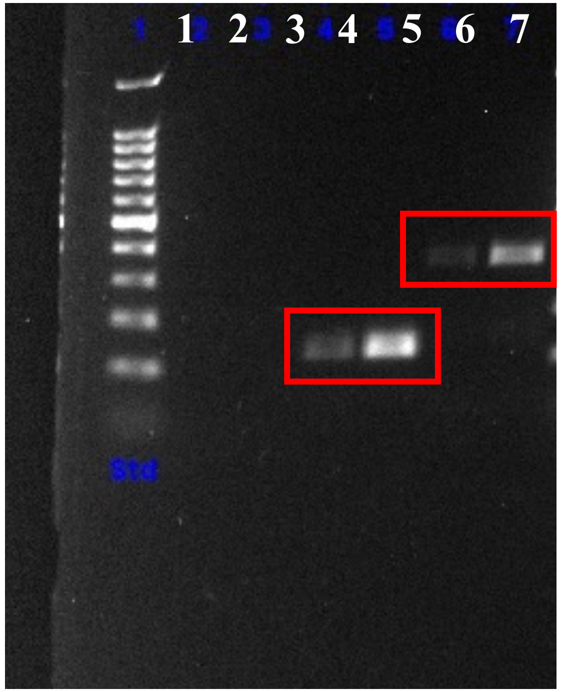

Nitrate transporter
Lane 4: Control
Lane 5: Inoculated

Chloride transporter
Lane 6: Control
Lane 7: Inoculated

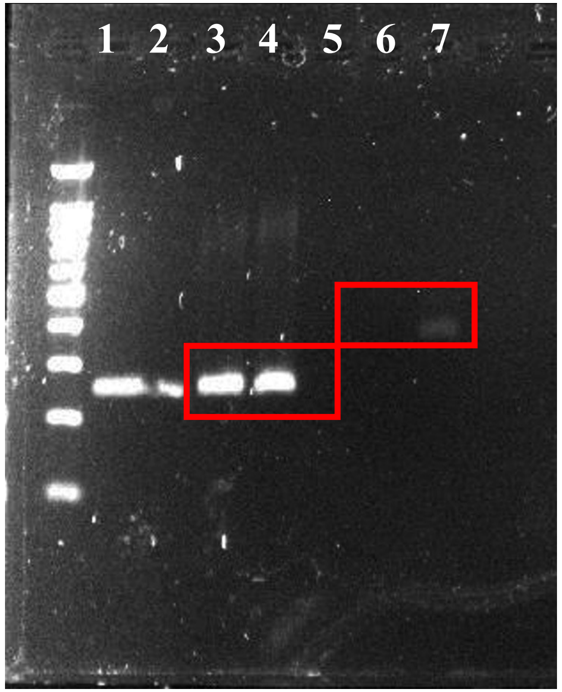

Cyclophilin
Lane 3: Control
Lane 4: Inoculated

Nitrate reductase
Lane 6: Control
Lane 7: Inoculated

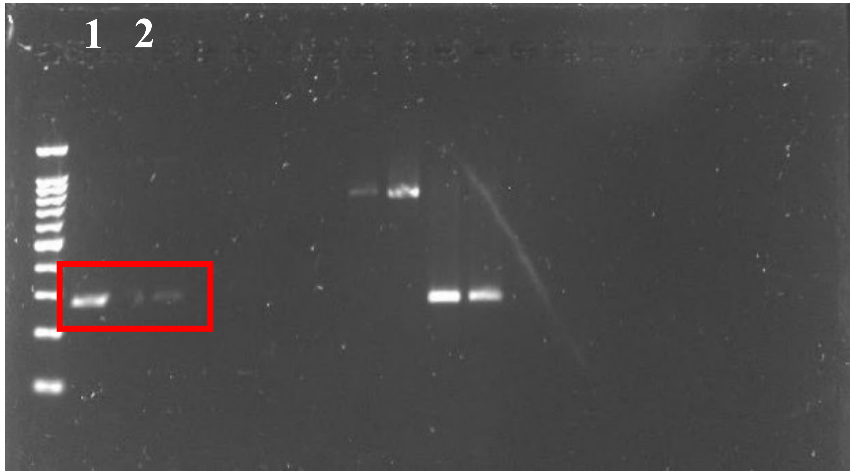

WRKY48
Lane 1: Control
Lane 2: Inoculated

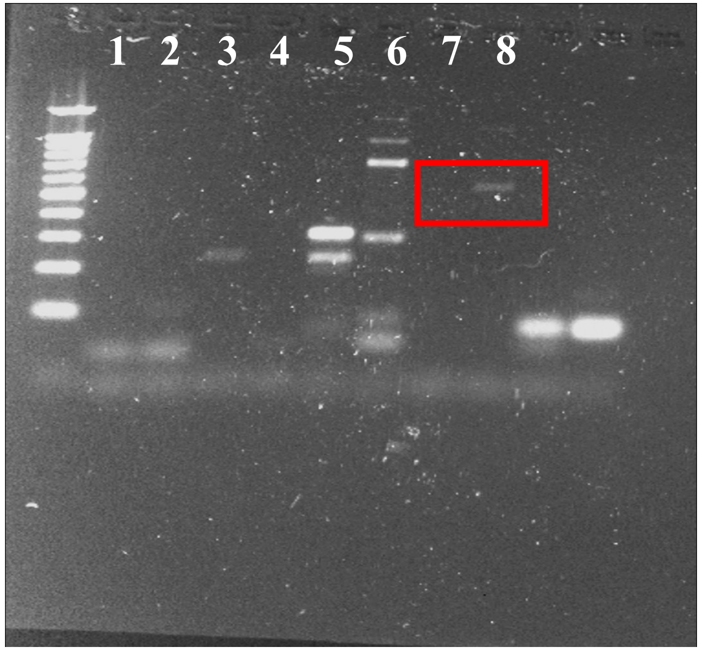

High affinity nitrate transporter
Lane 7: Control
Lane 8: Inoculated

Supplementary Figure 2: This figure contains the original, unedited gel images used in figures in this study.

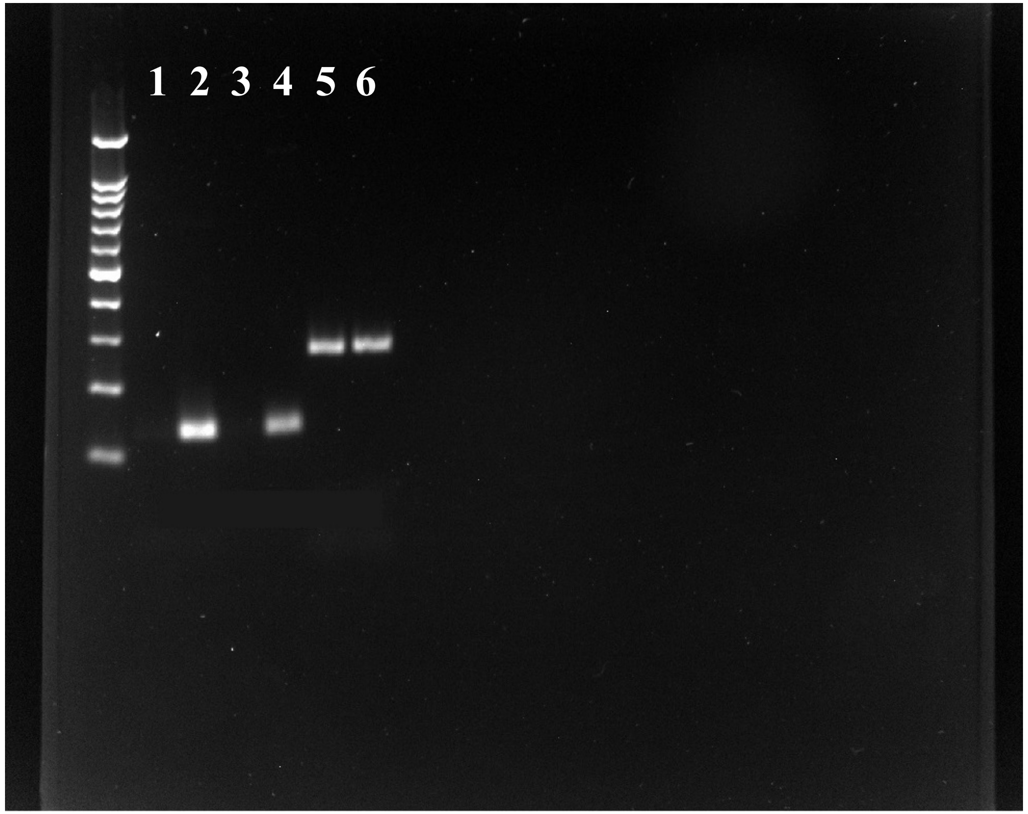

nifH
Lane 1: Uninoculated
Lane 2: Inoculated

gfp
Lane 3: Uninoculated
Lane 4: Inoculated

Cyclophilin
Lane 5: Uninoculated
Lane 6: Inoculated

Gel used for Supplementary Figure 1
